# Duvelisib attenuates bleomycin‐induced pulmonary fibrosis via inhibiting the PI3K/Akt/mTOR signalling pathway

**DOI:** 10.1111/jcmm.17665

**Published:** 2023-01-18

**Authors:** Xiaohe Li, Xiaoyang Ma, Yang Miao, Jianwei Zhang, Buri Xi, Wenqi Li, Qianyi Zhang, Li Chen, Yue Yang, Hongli Li, Luqing Wei, Honggang Zhou, Cheng Yang

**Affiliations:** ^1^ State Key Laboratory of Medicinal Chemical Biology, College of Pharmacy and Tianjin Key Laboratory of Molecular Drug Research Nankai University Tianjin China; ^2^ Tianjin Key Laboratory of Molecular Drug Research Tianjin International Joint Academy of Biomedicine Tianjin China; ^3^ Department of Respiratory and Critical Care Medicine Tianjin Beichen Hospital Tianjin China

**Keywords:** Duvelisib, myofibroblasts, PI3K/Akt/mTOR signalling pathway, pulmonary fibrosis

## Abstract

Idiopathic pulmonary fibrosis (IPF) is a chronic progressive interstitial lung disease that seriously threatens the health of patients. The pathogenesis of IPF is still unclear, and there is a lack of effective therapeutic drugs. Myofibroblasts are the main effector cells of IPF, leading to excessive deposition of extracellular matrix (ECM) and promoting the progression of fibrosis. Inhibiting the excessive activation and relieving autophagy blockage of myofibroblasts is the key to treat IPF. PI3K/Akt/mTOR pathway plays a key regulatory role in promoting fibroblast activation and autophagy inhibition in lung fibrosis. Duvelisib is a PI3K inhibitor that can simultaneously inhibit the activities of PI3K‐δ and PI3K‐γ, and is mainly used for the treatment of relapsed/refractory chronic lymphocytic leukaemia (CLL) and small lymphocytic lymphoma tumour (SLL). In this study, we aimed to examine the effects of Duvelisib on pulmonary fibrosis. We used a mouse model of bleomycin‐induced pulmonary fibrosis to evaluate the effects of Duvelisib on pulmonary fibrosis in vivo and further explored the potential pharmacological mechanisms of Duvelisib in lung fibroblasts in vitro. The in vivo experiments showed that Duvelisib significantly alleviated bleomycin‐induced collagen deposition and improved pulmonary function. In vitro and in vivo pharmacological experiments showed that Duvelisib dose‐dependently suppressed lung fibroblast activation and improved autophagy inhibition by inhibiting the phosphorylation of PI3K, Akt and mTOR. Our results indicate that Duvelisib can alleviate the severity of pulmonary fibrosis and provide potential drugs for the treatment of pulmonary fibrosis.

## 1 INTRODUCTION

Idiopathic pulmonary fibrosis (IPF) is a chronic, progressive pulmonary interstitial disease characterized by diffuse alveolar inflammation and alveolar structural disorder, which eventually leads to pulmonary interstitial fibrosis.^1^ As the most common and serious type of idiopathic interstitial pneumonia, it is characterized by the destruction of normal alveolar tissue, excessive deposition of extracellular matrix (ECM) and structural changes, resulting in reduced pulmonary ventilation, blocked gas exchange and irreversible decline of pulmonary function.^2^ IPF seriously threatens the health of patients, and can eventually lead to respiratory failure and even death.^3^

PI3K/Akt signal pathway regulates cell survival, proliferation, differentiation, cell metabolism and plays an important role in the pathogenesis of pulmonary fibrosis.^4^, ^5^, ^6^ When PI3K is activated, the p85 regulatory subunit of PI3K is recruited to the site adjacent to the plasma membrane, and the P110 subunit converts the substrate PIP2 to PIP3 by binding with p85 subunit. PIP3 can combine with Akt to phosphorylate threonine phosphorylation site (Thr308) and serine phosphorylation site (Ser473) of Akt protein. Then Akt can activate downstream signal molecules, such as mammalian target of rapamycin (mTOR), forkhead‐type transcription factor (Fox protein) and Bcl‐2 anti‐apoptotic protein.^7^ PTEN, the main inhibitor of PI3K/Akt signalling, is down regulated in pulmonary fibrosis in patients with IPF.^8^ In vitro model confirmed that PTEN low expression fibroblasts showed higher Akt phosphorylation level, so they were resistant to fibroblast apoptosis caused by collagen matrix contraction. Low activity autophagy has become an important cause of IPF.^9^, ^10^ PI3K/Akt/mTOR signal pathway is the main regulatory pathway in this process.^11^ Therefore, PI3K/Akt/mTOR pathway is closely related to autophagy in the process of IPF and plays an important role in IPF.

Duvelisib is an oral dual inhibitor of PI3K‐δ and PI3K‐γ. PI3K‐δ/γ inhibition may directly inhibit malignant T‐cell growth, making Duvelisib a promising candidate for patients with peripheral (PTCL) or cutaneous (CTCL) T‐cell lymphoma.^12^ Because PI3K/Akt/mTOR signalling pathway is closely related to autophagy in the process of IPF, we hypothesized that Duvelisib may have a therapeutic role in pulmonary fibrosis. Therefore, in this study, we verified the effect of Duvelisib in pulmonary fibrosis by in vivo and in vitro experiments.

## 2 MATERIALS AND METHODS

### 2.1 Materials

Recombinant human transforming growth factor‐β1 (TGF‐β1) was purchased from Peprotech. Antibodies against fibronectin, P‐mTOR/mTOR, P62, LC3I/II were purchased from Cell Signalling Technology. Antibodies against α‐SMA, P‐PI3K/PI3K, P‐Akt/Akt, GAPDH were obtained from Affinity Biosciences. Goat pAbs against Rb IgG (HRP) and Rb pAbs against Ms IgG were obtained from ImmunoWay (China). TRIzol reagent was acquired from Ambion Life Technology (China). DEPC‐treated H2O and RNAse Away H_2_O were purchased from Life Technologies. Fastking gDNA Dispelling RT SuperMix was purchased from Tiangen. UNICON® qPCR SYBR Green Master Mix and liposomal transfection reagents were purchased from Yevsen. The Masson's trichrome staining kit was obtained from Solarbio (China).

### 2.2 Cell culture

Mouse lung fibroblasts (Mlg, ATCC) and mouse primary lung fibroblasts (PPF) were maintained in DMEM (KeyGEN BioTECH) with 10% FBS. Primary mouse lung fibroblast were maintained in DMEM (KeyGEN BioTECH) with 10% FBS. All cells were incubated with 5% CO_2_ at 37°C.

### 2.3 Animals

Male C57BL/6 mice (6–8 weeks old, 20–25 g) were purchased from the Weitong Lihua Experimental Animal Technology Co. Ltd. The animal experimental protocol was performed in accordance with the Health Guide for Care from the National Institutes (NIH Publications no. 85‐23, revised 1996). All animal care and experimental procedures complied with guidelines approved by the Institutional Animal Care and Use Committee (IACUC) of Nankai University (permit no. SYXK 2019‐0001).

### 2.4 BLM administration

A mouse pulmonary fibrosis model was established by intratracheal BLM administration as described previously.^13^ Briefly, mice were intratracheally administered BLM (2 U) dissolved in physiological saline (0.9% NaCl). For pulmonary fibrosis models, 48 mice were randomly separated into six groups: (1) NaCl group, (2) BLM group (3) the positive control group (BLM + Nintedanib, 100 mg/kg), (4) the low‐dose Duvelisib group (BLM + Duvelisib, 12.5 mg/kg), (5) the middle‐dose Duvelisib group (BLM + Duvelisib, 25 mg/kg), (6) the high‐dose Duvelisib group (BLM + Duvelisib, 50 mg/kg). The mice in the Duvelisib groups were intraperitoneally injected daily with 12.5 , 25 or 50 mg/kg Duvelisib, which was suspended in CMC‐Na, while mice in the control and BLM groups received equal volumes of vehicle. Mice in the positive control group were intragastrically administered 100 mg/kg nintedanib daily. Carboxymethyl cellulose sodium (CMC‐Na) is a common solvent for animal administration. We dissolved 2 g of sodium carboxymethyl cellulose in 398 ml of pure water to obtain a CMC‐Na solution with a concentration of 0.5%. Then we used 0.5% CMC‐Na as the solvent to prepare different concentrations of Duvelisib and Nintedanib. Both drugs can be well dissolved in 0.5% CMC Na. The mice were sacrificed on the 14th day after BLM administration for subsequent analysis.

### 2.5 Hydroxyproline assay

Collagen content in lung homogenate was determined as previously described.^13^ In brief, the right lung of the mouse was isolated and put into 5 ml ampoules and dried. 3 ml of 6 M hydrochloric acid was added into each ampoule. After hydrolyzed, the PH is adjusted to 6.5–8.0. Then the samples were filtered and 1 × PBS volume was fixed to 10 ml. The samples were processed according to the hydroxyproline assay kit. The HYP analysis was carried out basically according to the manufacturer's instructions, each sample 200 μl was transferred into 96‐well plate and divided into 3 reproductions. Absorbance was determined at 577 nm.

### 2.6 Histological examination

The left lung was fixed in 10% formalin, dehydrated and embedded in paraffin. Tissue sections with a thickness of 4 μM were incubated for 4 h at 60°C and stained with haematoxylin and eosin (H&E) and Masson's trichrome. Images were analysed by Image‐Pro Plus version 6.0 to demarcate the entire lung area and automatically calculate the total pixel Pw of the region and then calculate the total pixel Pf of the fibrotic region (fibrosis ratio = fibrotic area total pixel Pf/total lung total pixel Pw).

### 2.7 Pulmonary function testing

After the mice were anaesthetised, the trachea was exposed, and a tracheal cannula was placed and fixed. The mice were transferred to a plethysmography chamber for pulmonary function analysis using an Anires2005 system according to the manufacturer's instructions (Beijing Biolab).

### 2.8 Cell viability analysis

Cell viability was measured using 3‐(4,5‐dimethylthiazol‐2‐yl)‐2,5‐diphenyltetrazolium bromide (MTT) and CCK‐8 as previously described.^14^

### 2.9 EdU assay

Briefly, 1 × 10^5^ cells/well were plated in 24‐well plates with diameter a 14‐mm diameter coverslip. Duvelisib at various concentration was added to the wells for 24 h. Then, 40 μM 5‐ethynyl‐2′‐deoxyuridine (Invitrogen) was added to the wells for an additional 2 h. To fix and improve cell membrane permeability, 4% paraformaldehyde and 0.5% Triton X‐100 in PBS were applied for 15 min. Hoechst 33342 was used to label cell nuclei. A fluorescence microscope was used to view the signal.

### 2.10 Wound healing assay

Wound healing assays were performed as previously described.^13^

### 2.11 Transwell cell migration assay

Transwell assays can not only reveal cell migratory behaviours but also are used to observe variations in cellular morphology. Briefly, 1 × 10^5^ fibroblasts/ml were suspended in serum‐free DMEM medium, and 100 μl of the cell suspension was seeded on the top filter membrane and added different concentrations of Duvelisib and TGF‐β1, followed by incubation for 24 h.

DMEM medium containing 20% FBS is added into the lower chamber of 24‐well plate. he cells were fixed with 70% paraformaldehyde for 10 min. Then, a cotton‐tipped applicator was used to remove the remaining cells and paraformaldehyde from the top filter membrane. Next, 0.2% crystal violet was added to the low chamber and incubated for 15 min at 25°C. After staining, the remaining crystal violet was carefully removed from the top membrane using a cotton‐tipped swab, and the cells were gently washed with water. An inverted microscope was used to view the signal.

### 2.12 Quantitative real‐time PCR

The mRNA expression level of genes was determined by quantitative real‐time PCR (qRT‐PCR) using primers according to a previously described protocol.^15^ Primer pairs of target genes used were as follows:

α‐SMA (NM_001297715.1), 5′‐TGGGTGAACTCCATCGCTGTA‐3′ and 5′‐GTCGAA TGCAACAAGGAAGCC‐3′; Fibronectin (NM_001306132.1), 5′‐GTGCCCGGAATACGCATGTA‐3′ and 5′‐CTGGTGGACGGGATCATCCT‐3′; GAPDH (NM_008084), 5′‐AGGTCGGTGTGAACGGATTTG‐3′ and 5′‐GGGGTCGTTGATGGCAACA‐3′.

### 2.13 Western blot analysis

The proteins were extracted from lung tissues or cells following standard protocols, as described previously.^16^ Protein was extracted from lung tissue homogenates or cells using Radio‐Immunoprecipitation Assay (RIPA) lysis buffer containing Protease inhibitor cocktail (CT) and sodium fluoride (NaF). After electrophoresis and membrane transfer, the following primary antibodies were used to explore the western blot: GAPDH, α‐SMA, Fibronectin, P62, LC3I/II, P‐PI3K/PI3K, P‐Akt/Akt, P‐mTOR/mTOR. The secondary antibodies were goat anti‐rabbit or goat anti‐mouse horseradish peroxidase‐conjugated antibodies. The protein bands were visualized using an enhanced chemiluminescence system (Affinity Biosciences), GAPDH was used as loading control.

### 2.14 Immunofluorescence analysis

Mlg cells were seeded in a 24‐well chamber. At the end of the treatment, the cells were fixed with a 4% fixative solution for 15 min (Solarbio), permeabilized with 0.2% Triton X‐100 for 20 min, and incubated in 5% BSA for 30 min. Mlg cells were incubated overnight with α‐SMA primary antibodies. Then, the cells were incubated with FITC‐conjugated secondary antibodies. The cells were washed with PBST, and DAPI (Beyotime Biotechnology) was used to stain the nuclei. The fluorescence was examined with a confocal microscope (Nikon).

### 2.15 Immunohistochemical staining

Paraffin‐embedded lung tissue was dewaxed with xylene, and the sections were heated in a microwave oven with antigen‐fixing solution (0.01 M citrate buffer) for 20 min. After the sections were cooled to room temperature and blocked with an immunohistochemistry kit, the primary antibody was added and incubated at 4°C overnight. The primary antibodies were as follows: mouse anti‐α‐SMA (1:200 dilution), mouse anti‐fibronectin (1:200 dilution) and rabbit anti‐p62 (1:200 dilution). After being washed with PBST for three times, the tissue sections were incubated with the secondary antibody at room temperature for 1 h. Then the tissue sections were stained with a DAB substrate kit and haematoxylin. A neutral balsam was used to fix the sections, and an optical microscope was used to assess the expression of the proteins.

### 2.16 Data and statistical analysis

All statistical were performed using Graphpad prism 7.0 software as the means ± SD (GraphPad Software, Inc). All statistical comparisons were analysed by one‐way analysis of variance followed by the Tukey–Kramer test to identify significant differences between groups. *p* < 0.05 was considered to be statistically significant.

## 3 RESULTS

### 3.1 Duvelisib attenuates BLM‐induced pulmonary fibrosis in mice

**FIGURE 1:**
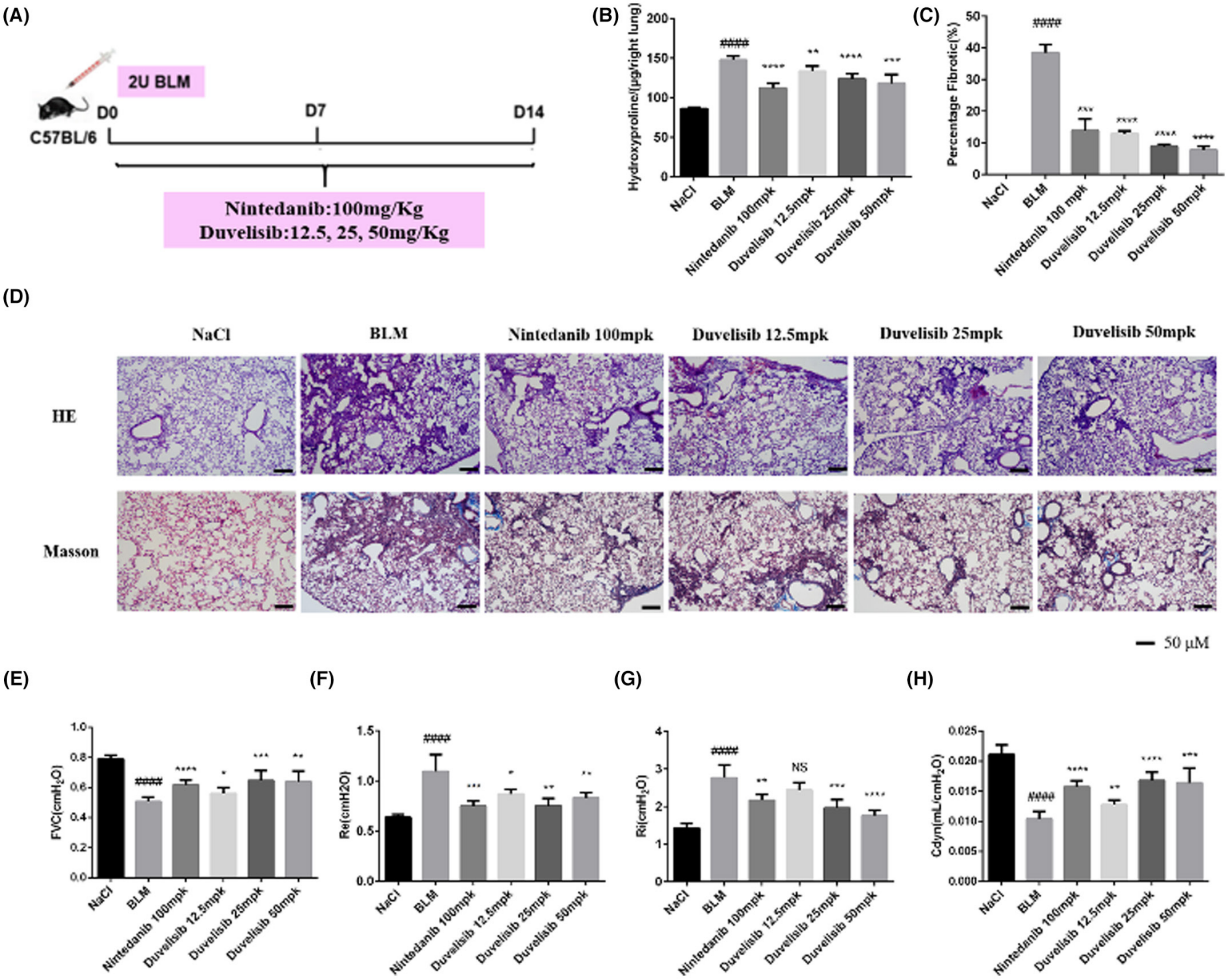
Duvelisib ameliorates BLM‐induced pulmonary fibrosis in mice. (A) Dosing regimen in BLM‐induced pulmonary fibrosis model. (B) Hydroxyproline contents of lung tissues in mice. (C) Statistics of lung fibrosis area among groups. (D) Lung tissue sections were stained with haematoxylin–eosin (H&E) and Masson Trichrome staining. (E) Forced vital capacity (FVC) of mice. (F) Expiratory resistance (Re) of mice. (G) Inspiratory resistance (Ri) of mice. (H) Dynamic compliance (Cydn) of mice. Data are shown as mean ± SD (*n* = 5). ##*p* < 0.01, ###*p* < 0.001, ####*p* < 0.0001 as compared with control group. *p < 0.05, **p < 0.01, ***p < 0.001, ****p < 0.0001 as compared with model group.

To determine the role of Duvelisib in pulmonary fibrosis in vivo, C57BL/6J mice were administered BLM (2 U), and then different doses of Duvelisib (12.5, 25 and 50 mpk) were administered for 14 days (day 1–day 14) (Figure 1A). Nintedanib (100 mpk) was used as a positive control. We found that the hydroxyproline level and percentage of fibrotic areas were significantly decreased in the Duvelisib‐treated group and that the inhibitory effect of the middle‐dose Duvelisib group (BLM + Duvelisib, 25 mpk) and the high‐dose Duvelisib group (BLM + Duvelisib, 50 mpk) were better than that of Nintedanib (Figure 1B,C). H&E and Masson's trichrome staining were performed to assess the degree of pulmonary fibrosis and showed the same results (Figure 1D). Lung function is a decisive factor in the diagnosis and treatment of pulmonary fibrosis in clinical trials. As shown in Figure 1 E–H, Duvelisib significantly improved lung function in BLM‐induced fibrotic mice, as seen by the increases in forced vital capacity (FVC) and dynamic compliance (Cdyn) and decreases in inspiratory resistance (Ri) and expiratory resistance (Re). These data revealed that Duvelisib could ameliorate BLM‐induced pulmonary fibrosis in mice.

### 3.2 Duvelisib suppresses TGF‐β1‐induced proliferation of lung fibroblasts

**FIGURE 2:**
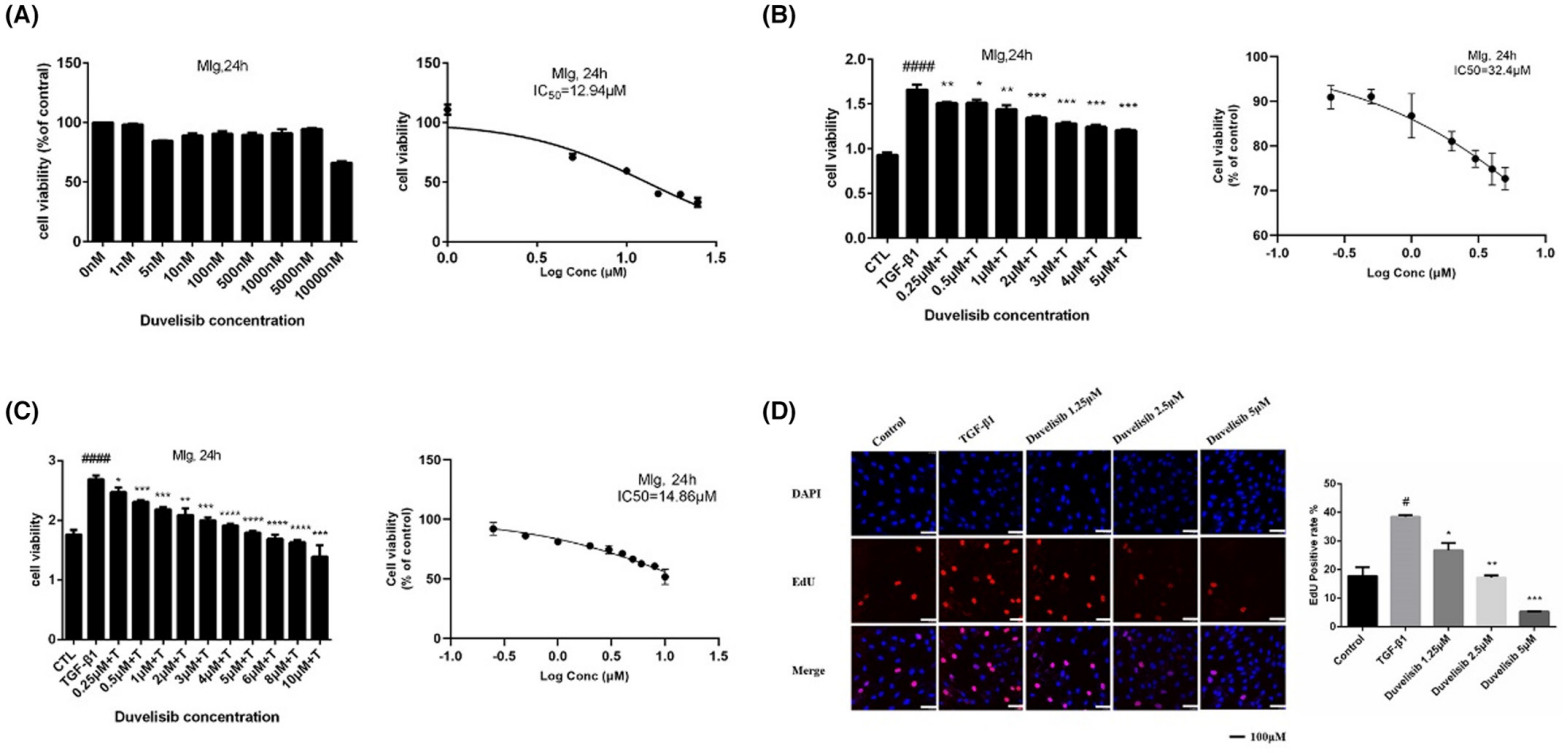
Duvelisib suppresses TGF‐β1‐induced proliferation of lung fibroblasts. (A) Cytotoxicity test of Mlg cells (MTT). Mlg cells were exposed to the indicated doses of Duvelisib for 24 h, IC50 = 12.94 μM (*n* = 4 per group). (B–D) Mlg cells were co‐cultured with TGF‐β1 (5 ng/ml) and indicated doses of Duvelisib for 24 h. (B) MTT assay was used to detect the effect of Duvelisib on cell proliferation. For TGF‐β1 stimulated Mlg cells, the IC50 value measured in MTT assay was 32.4 μM. (C) CCK‐8 assay was used to detect the effect of Duvelisib on cell proliferation. For TGF‐β1 stimulated Mlg cells, the IC50 value measured in CCK8 assay was 14.86 μM. (D) EdU assay was used to detect the effect of Duvelisib on cell proliferation (×40, scale bar = 100 μm). Data are shown as mean ± SD (*n* = 3). #*p* < 0.05, ####*p* < 0.0001 as compared with control group. *p < 0.05, **p < 0.01, ***p < 0.001, ****p < 0.0001 as compared with model group.

We first used MTT assay to verify whether Duvelisib could affect cell proliferation and survival of normal lung fibroblasts without TGF‐β1 stimulation. As shown in Figure 2A, Duvelisib had no significant toxic on normal lung fibroblasts at ≤5 μΜ and the IC50 was 12.94 μM. We then used TGF‐β1 to stimulate Mlg cells and co‐treated with Duvelisib for 24 h, then the proliferation of Mlg cells was detected by MTT assay (Figure 2B) and CCK‐8 assay (Figure 2C). The results indicated that Duvelisib could inhibit the proliferation of TGF‐β1‐actived lung fibroblasts in a concentration dependent manner since 0.25 μM, and the IC50 values measured in MTT and CCK8 experiments were 32.4 and 14.86 μM. Based on the above results, we finally chose three concentrations (1.25, 2.5 and 5 μM) under the conditions of no obvious toxicity and inhibition of cell proliferation. Then EdU analysis was used to verify the anti‐proliferative effect of Duvelisib, and the experimental results showed that EdU‐positive fibroblasts were decreased as the Duvelisib concentration increased (Figure 2D). In summary, these results indicated that Duvelisib could suppresses TGF‐β1‐induced proliferation of lung fibroblasts.

### 3.3 Duvelisib suppresses TGF‐β1‐induced migration of lung fibroblasts

**FIGURE 3:**
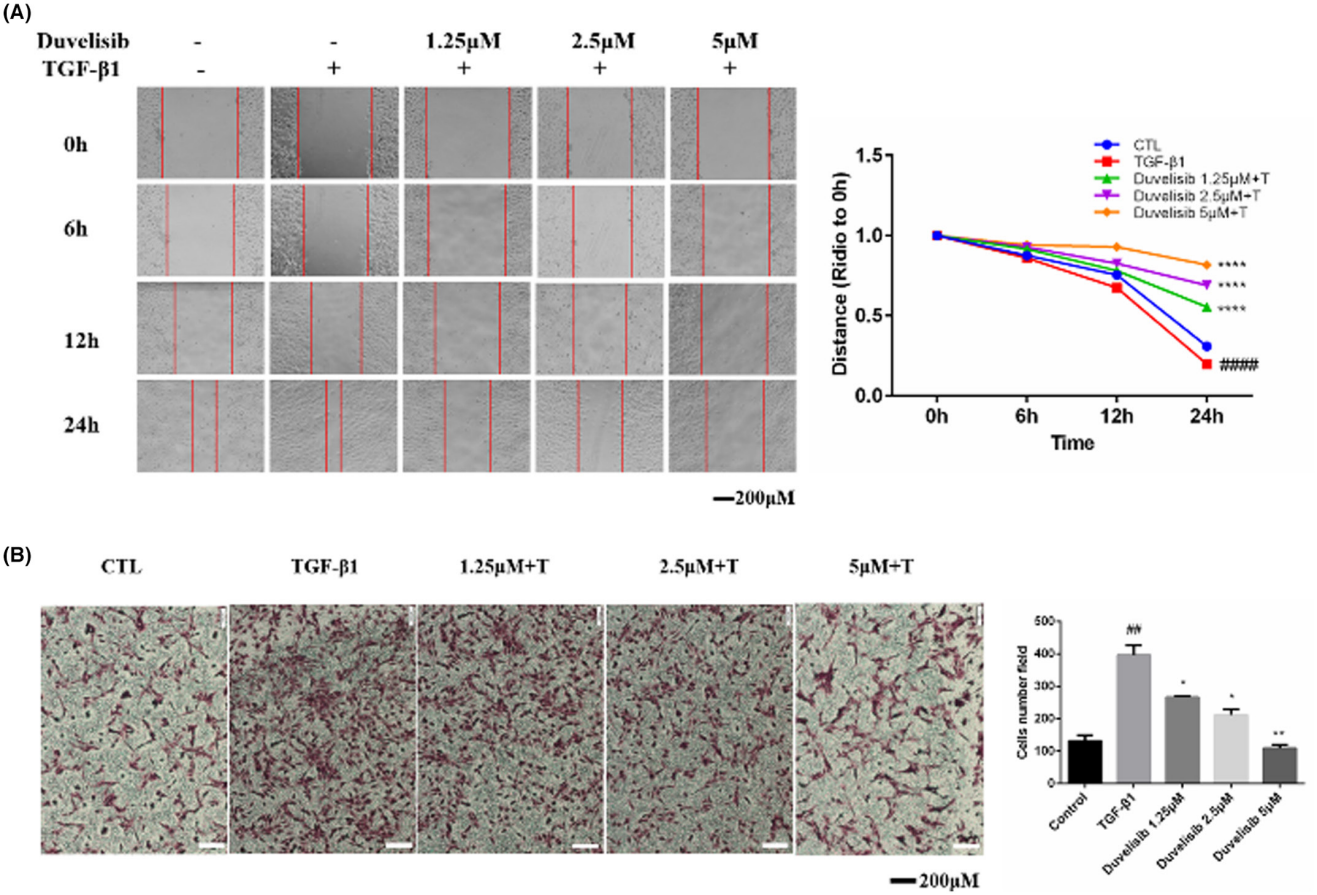
Duvelisib suppresses TGF‐β1‐induced migration of lung fibroblasts. (A) Wound healing assays of Mlg co‐cultured with TGF‐β1 (5 ng/ml) and Duvelisib (1.25, 2.5, 5 μM). The wound closure was photographed at 0, 6, 12, and 24 h post‐scratching. (B) Transwell assays of Mlg co‐cultured with TGF‐β1 (5 ng/ml) and Duvelisib (1.25, 2.5, 5 μM). The number of migrating cells was count at 24 h. Data are shown as mean ± SD (*n* = 3). ##*p* < 0.01, ####*p* < 0.0001 as compared with control group. *p < 0.05, **p < 0.01, ****p < 0.0001 as compared with model group.

Wound closure and transwell experiments were used to detect the inhibitory effect of Duvelisib on fibroblast migration. Wound closure assay results showed that Duvelisib inhibited fibroblast migration in a dose‐dependent manner (Figure 3A). Compared with that in the TGF‐β1 group, wound closure ability was reduced approximately 62% after treatment with 5 μM Duvelisib for 24 h (Figure 3A). Figure 3B demonstrates that fibroblast migration was suppressed, and cellular morphology was also altered with Duvelisib treatment. Most cells exhibited an irregular polygonal shape rather than a typical spindle shape after treatment with 5 μM Duvelisib for 24 h (Figure 3C). The number of migrated cells attached to the bottom membrane after treatment with 2.5 and 5 μM Duvelisib were reduced 47% and 73%, respectively, compared with the TGF‐β1 group. These data indicated that Duvelisib could suppresses TGF‐β1‐induced migration of lung fibroblasts.

### 3.4 Duvelisib attenuates TGF‐β1‐induced activation of lung fibroblasts

**FIGURE 4:**
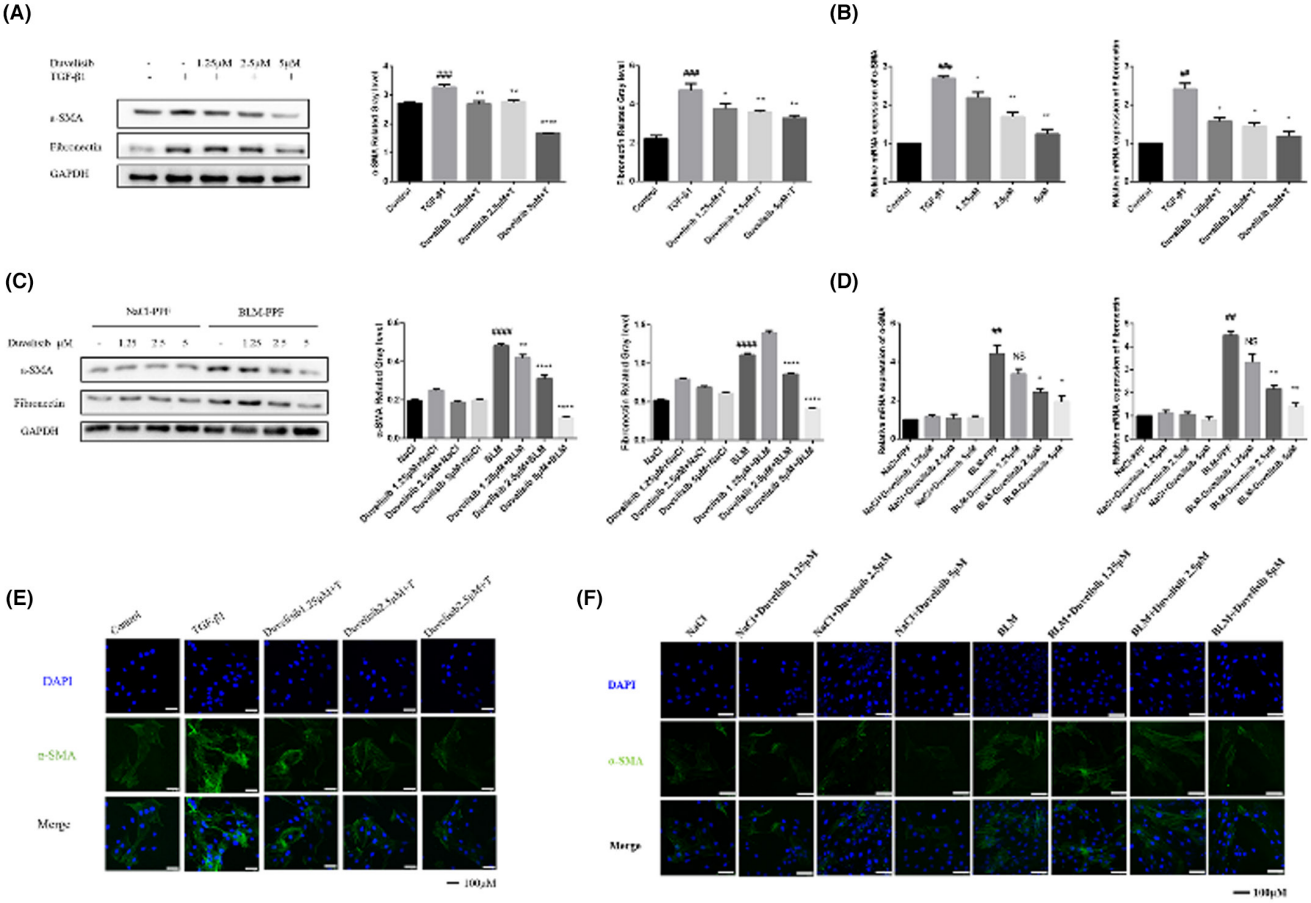
Duvelisib attenuates activation of lung fibroblasts in vitro. (A, B) Mlg cells were treated with TGF‐β1 (5 ng/ml) and Duvelisib (1.25, 2.5, 5 μM) for 24 h. The expression of α‐SMA and fibronectin were analysed by Western blot analysis (A) and Real‐time PCR (B) in Mlg cells. (C, D) NaCl/BLM‐PPF cells were treated with Duvelisib (1.25, 2.5, 5 μM) for 24 h. The expression of α‐SMA and fibronectin were analysed by Western blot analysis (C) and Real‐time PCR (D) in NaCl/BLM‐PPF cells. (E) Immunofluorescence staining of α‐SMA were performed on Mlg cells treated with/without TGF‐β1 (5 ng/ml) and/or Duvelisib (1.25, 2.5, 5 μM) for 24 h. (F) Immunofluorescence staining of α‐SMA were performed on NaCl/BLM‐PPF cells treated with Duvelisib (1.25, 2.5, 5 μM) for 24 h. Data are shown as mean ± SD (*n* = 3). ##*p* < 0.01, ###*p* < 0.001, ####*p* < 0.0001 as compared with control group. *p < 0.05, **p < 0.01, ****p < 0.0001 as compared with model group.

To further examine whether Duvelisib could inhibit the activation of TGF‐β1‐induced fibroblasts, we treated Mlg cells with TGF‐β1 (5 ng/ml) and Duvelisib (1.25, 2.5, and 5 μM) to assess the protein expression levels of the typical fibroblast activation marker α‐SMA and the extracellular matrix (ECM) proteins fibronectin. As shown in Figure 4A, Duvelisib dose‐dependently decreased TGF‐β1‐induced expression of the α‐SMA and fibronectin proteins. Similarly, Duvelisib treatment reduced the gene expression levels of α‐SMA and fibronectin in TGF‐β1‐stimulated fibroblasts (Figure 4B). PPF cells experiments also showed the same results (Figure 4C,D). Immunofluorescence assays were used to evaluate α‐SMA expression in Mlg cells and PPF cells. The results were consistent with the above (Figure 4 E,F). Accordingly, these results indicated that Duvelisib suppressed TGF‐β1‐induced myofibroblast differentiation and reduced ECM production in myofibroblasts.

### 3.5 Duvelisib enhances TGF‐β1‐inhibited autophagy in lung fibroblasts

**FIGURE 5:**
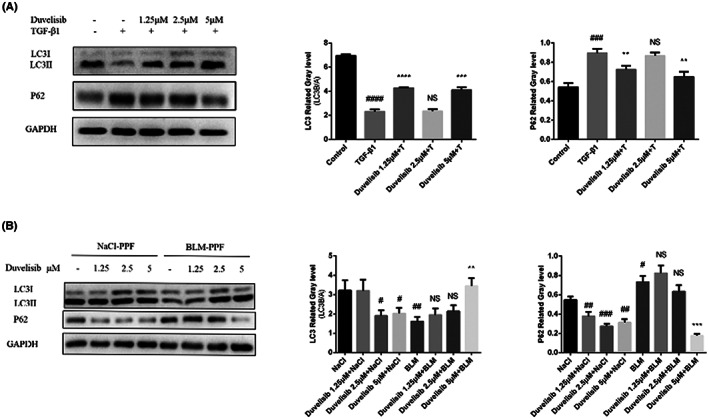
Duvelisib relieved blocked autophagy in lung fibroblasts in vitro. (A) The total protein of Mlg cells was extracted for Western blot to detect the protein expression level of autophagy markers LC3I, LC3II and p62. (B) The total protein of NaCl/BLM‐PPF cells was extracted for Western blot to detect the protein expression level of autophagy markers LC3I, LC3II and p62. GAPDH was used as a loading control in grayscale analysis. Data are shown as mean ± SD (*n* = 3). #*p* < 0.05, ##*p* < 0.01, ###*p* < 0.001, ####*p* < 0.0001 as compared with control group. **p < 0.01, ***p < 0.001, ****p < 0.0001 as compared with model group.

In the process of autophagosome formation, a large number of proteins are required to participate in the formation of autophagosome membrane, such as LC3 and p62. The ratio of LC3II/I can estimate the level of autophagy.^17^ P62 is an autophagy substrate in cells and the higher the expression of P62, the more serious the autophagy damage.^18^ We examined the effect of Duvelisib on fibroblasts with blocked autophagy and the experimental results showed that Duvelisib could significantly increase the expression level of autophagy related protein LC3II and inhibit the expression of P62 in Mlg cells and BLM‐PPF cells (Figure 5). These results demonstrated that Duvelisib could relieve blocked autophagy in lung fibroblasts in vitro.

### 3.6 Duvelisib inhibits TGF‐β1‐induced activation of PI3K/Akt/mTOR signal pathway in lung fibroblasts

**FIGURE 6:**
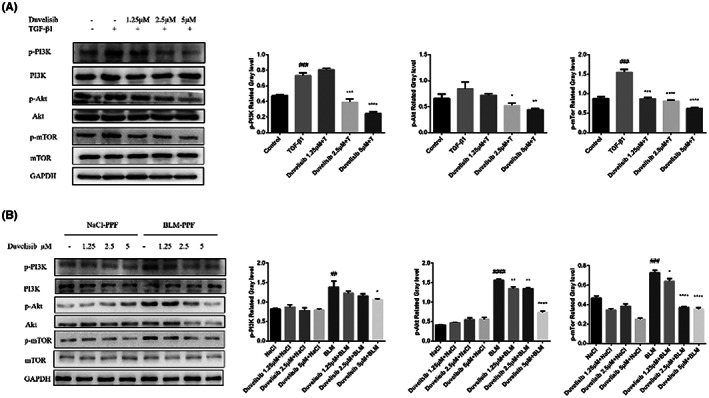
Duvelisib inhibited activation of PI3K/Akt/mTOR signalling pathway in lung fibroblasts in vitro. (A) Mlg cells were treated with TGF‐β1 (5 ng/ml) and Duvelisib (1.25, 2.5, 5 μM) for 3 h and the phosphorylation levels of PI3K, Akt and mTOR were analysed by Western blot. (B) NaCl/BLM‐PPF cells were treated with Duvelisib (1.25, 2.5, 5 μM) for 3 h and the phosphorylation levels of PI3K, Akt and mTOR were analysed by Western blot. GAPDH was used as a loading control in grayscale analysis. Data are shown as mean ± SD (*n* = 3). ##*p* < 0.01, ###*p* < 0.001, ####*p* < 0.0001 as compared with control group. *p < 0.05, **p < 0.01, ***p < 0.001, ****p < 0.0001 as compared with model group.

Because Duvelisib is a PI3K inhibitor and PI3K/Akt/mTOR pathway is closely related to fibroblasts activation and autophagy in the process of IPF, we detected the expression of three main related proteins of this signal pathway: PI3K, Akt, mTOR in Mlg and PPF cells by Western blotting. As shown in Figure 6A, Duvelisib significantly reduced TGF‐β1‐induced phosphorylation of PI3K, Akt and mTOR in Mlg cells. Moreover, the levels of phosphorylated PI3K, Akt and mTOR were examined in PPF and the results suggested that Duvelisib could decreased the phosphorylation levels of PI3K, Akt and mTOR in BLM‐PPF (Figure 6B). These results revealed that Duvelisib restrained the activation of PI3K/Akt/mTOR signalling in active fibroblasts.

### 3.7 Duvelisib attenuates BLM‐induced fibroblast activation and enhances autophagy in vivo

**FIGURE 7:**
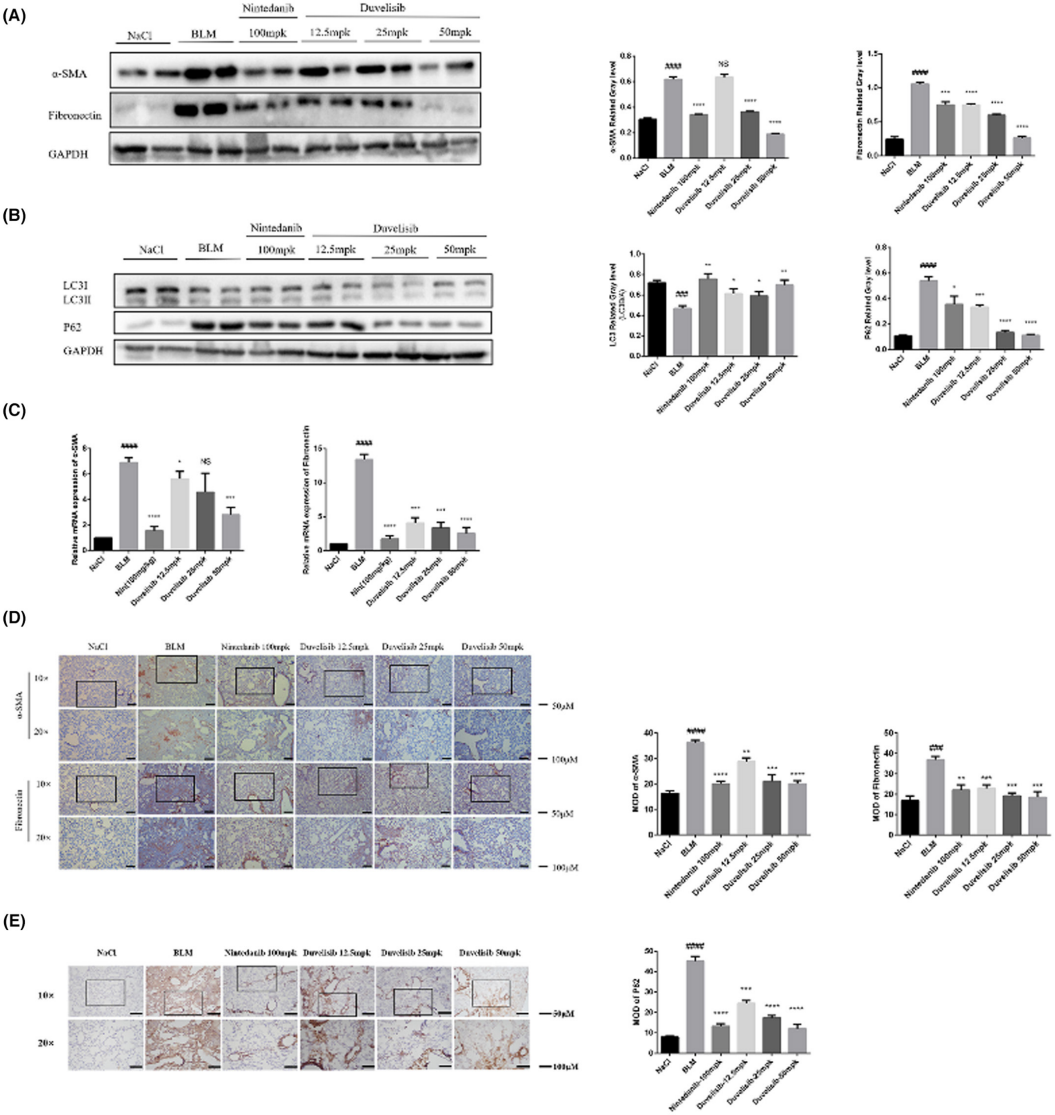
Duvelisib inhibited BLM‐induced activation and relieved autophagy blocked of fibroblasts in vivo. (A) Protein levels of α‐SMA and fibronectin were verified by Western blot in lung tissues. GAPDH was used as an internal reference in densitometric analysis. (B) Protein levels of LC3 II and p62 were verified by Western blot in lung tissues. (C) RT‐PCR was performed to detect mRNA levels of α‐SMA and fibronectin. (D–E) Immunohistochemical staining analysis of α‐SMA, fibronectin (D) and p62 (E) in the lung tissues. Data are shown as mean ± SD (*n* = 3). ##*p* < 0.01, ###*p* < 0.001, ####*p* < 0.0001 as compared with control group. **p < 0.01, ***p < 0.001, ****p < 0.0001 as compared with model group.

In order to further explore the effect of Duvelisib on BLM induced pulmonary fibrosis, we extracted RNA and protein from mouse lung tissues, carried out immunohistochemistry experiment on lung tissue sections, and determined expression of α‐SMA and fibronectin. The results showed that compared with NaCl group, the expression of α‐SMA and fibronectin of BLM group was significantly increased. After Duvelisib treatment, the expression of α‐SMA and fibronectin decreased significantly, and the inhibitory effect of Duvelisib in high‐dose group was equivalent to Nintedanib (Figure 7A,C). The immunohistochemistry experiment of α‐SMA and fibronectin showed the same results (Figure 7D). These results indicated that Duvelisib can inhibit the activation of fibroblasts in vivo. Additionally, the autophagy‐related proteins LC3 and p62 were detected by western blot, and their expression levels were improved by Duvelisib treatment in a dose‐dependent manner (Figure 7B). The immunohistochemistry experiment of p62 showed the same results (Figure 7E). These data indicated that Duvelisib could promote autophagy in vivo.

### 3.8 Duvelisib inhibits activation of PI3K/Akt/mTOR signal pathway in vivo

**FIGURE 8:**
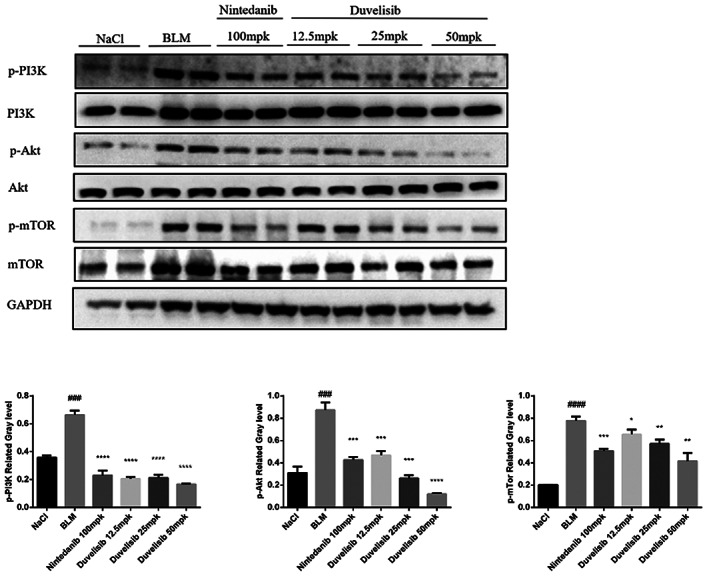
Duvelisib inhibited activation of PI3K/Akt/mTOR signalling pathway in lung fibroblasts in vivo. The phosphorylation levels of PI3K, Akt and mTOR in lung tissues were detected by Western blot. Data are shown as mean ± SD (*n* = 3). ###*p* < 0.001, ####*p* < 0.0001 as compared with control group. *p < 0.05, ***p < 0.001, ****p < 0.0001 as compared with model group

We have demonstrated that Duvelisib attenuated pulmonary fibrosis through inhibiting the phosphorylation of PI3K/AKT/mTOR signal pathway in vitro. To further verified the mechanism of the anti‐fibrosis of Duvelisib in vivo, we performed Western blot on the lung tissues of BLM‐injured mice. As shown in Figure 8, BLM induced the phosphorylation of PI3K, AKT and mTOR while had no influence on the total PI3K, AKT and mTOR. On the contrary, Duvelisib administration inhibited the expression of the phosphorylation of PI3K, AKT and mTOR induced by BLM, this result was consistent with the in vitro experiments.

## 4 DISCUSSION

Idiopathic pulmonary fibrosis is a fatal chronic interstitial lung disease that is usually triggered by recurrent, multifocal lung parenchymal injury.^19^ Patients with IPF only have a median survival of 2.8 years and often die of respiratory failure.^2^ The efficacies of drugs for IPF are not high enough and cannot successfully reverse the progression and final outcome of IPF.^20^ Compared with the risk of time‐consuming, high cost, and low success rate of new drug development, the redevelopment of marketed drugs can shorten the time of clinical trials, reduce costs, and improve the success rate. Duvelisib, a PI3K‐targeted inhibitor that inhibits p110δ and p110γ activity, has been approved for the treatment of relapsed/refractory chronic lymphocytic leukaemia (CLL) and small lymphocytic lymphoma (SLL).^21^ In this study, we used in vivo and in vitro disease models to explore the efficacy and mechanism of Duvelisib on pulmonary fibrosis. The results found that Duvelisib could regulate lung fibroblasts proliferation, migration, activation and autophagy by inhibiting the PI3K/Akt/mTOR signalling pathway, thereby attenuating pulmonary fibrosis.

Bleomycin (BLM) is a clinical chemotherapeutic drug used to treat malignant tumours, and high‐dose administration can cause lung injury and fibrosis.^22^ Therefore, BLM is widely used to induce lung fibrosis in animals.^23^, ^24^ Intratracheal administration of BLM caused lung inflammation in the first week and pulmonary fibrosis in the second and third weeks.^25^ Pathological changes in BLM‐induced pulmonary fibrosis include myofibroblast activation and extracellular matrix deposition.^26^ In our study, we established BLM‐induced pulmonary fibrosis model in mice and chose 14 days as the time point to measure lung fibrosis parameters. At this time, the animals developed extensive fibrosis and had a lower mortality than later (day 21), and the quantitative analysis of fibrotic parameters demonstrated less variability at 14 days compared to 21 days.^27^ The results of animal model experiments showed that Duvelisib could improve the decline of pulmonary function and reduce collagen deposition in the lung tissue, thereby attenuate bleomycin‐induced pulmonary fibrosis in mice.

Myofibroblasts are currently recognized as the main effector cells of IPF, with significantly enhanced proliferation and ECM production ability, forming a large number of lesions in the lung interstitium.^28^ In addition to hyperactivation, myofibroblasts also exhibit a phenotype of insufficient autophagy, which is also involved in regulating ECM formation.^9^, ^29^ Studies have shown that increasing the autophagic clearance of type 1 collagen by lung fibroblasts can reduce the invasiveness of IPF fibroblasts.^30^ Different studies have also confirmed that autophagy activity is reduced in the lung tissues of IPF patients.^31^, ^32^ PI3K/Akt/mTOR is a classic autophagy signalling pathway, and studies have shown that the PI3K/Akt/mTOR signalling pathway is closely related to pulmonary fibrosis.^33^ Changes in the PI3K/Akt/mTOR signalling axis enable IPF fibroblasts to maintain a pathological phenotype of collagen overproduction by inhibiting autophagy.^34^ Decreased expression of FoxO3a, a direct target of Akt, inhibits the production of the autophagy marker LC3B on the collagen matrix, thereby inhibiting the autophagic response to collagen in IPF fibroblasts.^35^ Inhibition of mTOR activation stimulates autophagy, which is characterized by increased beclin1 and LC3 levels and autophagosome formation.^36^ Novel small‐molecule inhibitors targeting the PI3K/mTOR pathway have recently entered clinical trials for the treatment of IPF.^37^ Duvelisib is a PI3K‐targeted inhibitor that simultaneously inhibits the activities of PI3K‐δ and PI3K‐γ.^37^ In previous studies, it was generally believed that p110α and p110β of PI3K were widely distributed, while p110δ and p110γ were mainly expressed in lymphocytes. However, studies by Enrico Conte et al. showed that both p110δ and p110γ were expressed in human lung fibroblasts, and blocking the activities of p110δ and p110γ could also inhibit Akt phosphorylation, α‐SMA expression and collagen deposition.^6^ In this study, we proved that Duvelisib could inhibit the activation of PI3K/Akt/mTOR signalling pathway in vitro and in vivo, then inhibits the proliferation, migration and activation of lung fibroblasts, alleviates the blockage of autophagy in fibroblasts, and thus attenuates the progression of pulmonary fibrosis.

In conclusion, our study showed that Duvelisib has a therapeutic effect in an animal model of bleomycin‐induced pulmonary fibrosis, indicating that Duvelisib is promising as a potential drug for the treatment of IPF.

## AUTHOR CONTRIBUTIONS

**Xiaohe Li:** Investigation (equal); methodology (equal); project administration (lead); validation (equal); writing – original draft (lead); writing – review and editing (lead). **Xiaoyang Ma:** Data curation (equal); formal analysis (equal); methodology (equal); software (equal); validation (equal); visualization (equal). **Yang Miao:** Data curation (equal); investigation (equal); methodology (equal); supervision (equal); validation (equal); writing – original draft (equal). **Jianwei Zhang:** Data curation (equal); validation (equal). **Buri Xi:** Formal analysis (equal); methodology (equal); software (equal); validation (equal). **Wenqi Li:** Conceptualization (equal); methodology (equal); resources (equal); supervision (equal). **Qianyi Zhang:** Data curation (equal); methodology (equal); software (equal); validation (equal). **Li Chen:** Data curation (equal); methodology (equal); validation (equal); visualization (equal). **Yue Yang:** Data curation (equal); investigation (equal); software (equal); validation (equal). **Hongli Li:** Investigation (equal); methodology (equal); supervision (equal); validation (equal). **Luqing Wei:** Data curation (lead); methodology (lead); project administration (equal); resources (lead); validation (equal). **Honggang Zhou:** Conceptualization (lead); data curation (lead); resources (equal); supervision (equal). **Cheng Yang:** Conceptualization (lead); funding acquisition (lead); resources (lead); supervision (lead); visualization (lead).

## FUNDING INFORMATION

This study was supported by The Shenzhen Science and Technology Program (grant JCYJ202103241220060170), Tianjin Science and Technology Project (grant 20JCYBJC00600) and The Foundation of Organ Fibrosis Druggability Joint Research Centre of Nankai and Guokaixingcheng (grant 735‐F1040051).

## CONFLICT OF INTEREST

The authors declare no conflict of interest.

## INSTITUTIONAL REVIEW BOARD STATEMENT

All animal care and experimental procedures complied with guidelines approved by the Institutional Animal Care and Use Committee (IACUC) of Nankai University (project code: SCXK 2019‐0001, date of approval: 14 January 2019; permit no. SYXK 2021‐0001). Animal studies are reported in compliance with the ARRIVE guidelines (Kliment and Oury, 2010; McGrath and Lilley, 2015).

## Data Availability

No data are available in this study.
